# Diabetes mellitus and risk of injury requiring medical care: a nationwide population-based matched cohort study in Taiwan

**DOI:** 10.1186/s40621-026-00703-3

**Published:** 2026-08-05

**Authors:** Yao-Ching Huang, Ho-Tsung Hsin, Wei-Ting Lin, Tsu-Hsuan Weng, Chi-Hsiang Chung, Shi-Hao Huang, Bing‑Long Wang, Pi-Ching Yu, Pei-Yeh Yu, Li-Yun Fann, Wu-Chien Chien

**Affiliations:** 1 Department of Chemical Engineering and Biotechnology, National Taipei University of Technology (Taipei Tech), Taipei, 10608 Taiwan; 2 Department of Medical Research, Tri-Service General Hospital, National Defense Medical University, Taipei, 11490 Taiwan; 3 Graduate Institute of Public Health, College of Public Health, National Defense Medical University, Taipei, 11490 Taiwan; 4 Cardiovascular Intensive Care Unit, Department of Critical Care Medicine, Far-Eastern Memorial Hospital, New Taipei City, 10602 Taiwan; 5 Department of Education, Taipei City Hospital, Taipei, 10684 Taiwan; 6 Taiwanese Injury Prevention and Safety Promotion Association (TIPSPA), Taipei, 11490 Taiwan; 7 Graduate Institute of Medicine, College of Medicine, National Defense Medical University, Taipei, 11490 Taiwan; 8 Department of Anesthesiology, Taipei City Hospital Ren Ai branch, Taipei, 10684 Taiwan; 9 Department of Nursing, Taipei Tzu Chi Hospital, Buddhist Tzu Chi Medical Foundation, New Taipei City, 231016 Taiwan; 10 Department of Nursing, College of Nursing, Tzu Chi University, Hualien City, 97074 Taiwan; 11 Department of Nurse-Midwifery and Women Health, National Taipei University of Nursing and Health Sciences, Taipei city hospital, Taipei, 10684 Taiwan; 12 Institute of Biology and Anatomy, College of Medicine, National Defense Medical University, Taipei, 114201 Taiwan; 13 Graduate Institute of Life Sciences, College of Biomedical Sciences, National Defense Medical University, Taipei, 11490 Taiwan

**Keywords:** Diabetes mellitus (DM), Injury, Emergency department visit, Hospitalization, Propensity score matching (PSM)

## Abstract

**Background:**

Diabetes mellitus (DM) is associated with multiple microvascular and macrovascular complications; however, its association with clinically significant injury requiring medical care remains incompletely understood. This study aimed to investigate the association between DM and subsequent risk of injury requiring emergency department visits or hospitalization using a nationwide population-based cohort.

**Methods:**

We conducted a nationwide matched cohort study using Taiwan’s National Health Insurance Research Database. Adults with newly diagnosed DM were identified and matched with individuals without DM at a 1:3 ratio using propensity score matching. The propensity score model incorporated demographic characteristics, socioeconomic factors, baseline comorbidities, healthcare utilization, and overall disease burden. Covariate balance after matching was assessed using standardized mean differences (SMDs), with SMD < 0.10 indicating adequate balance. The primary outcome was clinically significant injury requiring emergency department visits or hospitalization. Multivariable Cox proportional hazards models with robust variance estimation were used to evaluate the association between DM and injury risk.

**Results:**

After propensity score matching, 31,424 individuals with DM and 94,272 matched individuals without DM were included. All baseline covariates achieved adequate balance after matching, with standardized mean differences (SMDs) <0.10. During follow-up, clinically significant injury requiring emergency department visits or hospitalization occurred more frequently among individuals with DM than among those without DM. In multivariable Cox proportional hazards models accounting for the matched cohort design, DM was independently associated with an increased risk of clinically significant injury (adjusted hazard ratio [aHR], 1.645; 95% confidence interval [CI], 1.202–2.015; p<0.001). The association remained robust in sensitivity analyses and was consistent across subgroup analyses stratified by age and sex, with no significant evidence of effect modification.

**Conclusion:**

In this nationwide population-based matched cohort study, DM was associated with an increased risk of clinically significant injury requiring emergency or inpatient care. These findings highlight the importance of incorporating injury risk assessment into comprehensive diabetes management, particularly among individuals with increased vulnerability due to age, diabetes-related complications, or functional impairment. Further studies incorporating detailed clinical information, including glycemic control and diabetes severity, are warranted to clarify the mechanisms underlying this association.

**Supplementary Information:**

The online version contains supplementary material available at https://doi.org/10.1186/s40621-026-00703-3.

## Introduction

Diabetes mellitus (DM) is a chronic metabolic disorder characterized by persistent hyperglycemia and represents a major global public health burden [1, 2]. The prevalence of DM has increased steadily over recent decades, with a growing proportion of affected individuals in low- and middle-income countries [3]. In Taiwan, DM is among the leading chronic diseases and contributes substantially to morbidity, mortality, and healthcare utilization [4, 5]. Complications of DM involve multiple organ systems, including cardiovascular, renal, neurological, and ophthalmologic systems, resulting in long-term functional impairment and increased healthcare demand [6, 7].

Injuries are a major cause of morbidity and preventable mortality worldwide [8]. Clinically significant injuries, particularly those requiring emergency department visits or hospitalization, represent a substantial healthcare burden and include traffic-related injuries, falls, fractures, burns, and poisoning [9, 10]. These injury events are associated with increased disability, prolonged hospitalization, and significant economic costs [11]. In Taiwan, accidental injury remains a leading cause of death and a significant public health concern across all age groups [12].

Emerging evidence suggests that patients with DM may have an increased risk of injury compared with non-diabetic populations [13–15]. Several biological and clinical mechanisms have been proposed, including hypoglycemia, peripheral neuropathy, impaired vision, autonomic dysfunction, sarcopenia, and polypharmacy, all of which may contribute to impaired balance and increased susceptibility to trauma [16, 17]. In addition, comorbid conditions commonly associated with DM, such as cardiovascular disease and chronic kidney disease, may further increase vulnerability to injury events [18].

However, existing epidemiological evidence remains inconsistent due to several methodological limitations. First, many prior studies have used broad or non-specific injury definitions, often including minor outpatient-treated injuries, which may lead to overestimation of incidence and reduced clinical interpretability [19]. Second, differential healthcare utilization among patients with chronic diseases such as DM may introduce detection bias, potentially inflating observed associations [20]. Third, few large-scale population-based studies have applied rigorous propensity score matching with formal covariate balance assessment using standardized mean differences (SMDs), limiting comparability between exposure groups.

Therefore, there remains a need for a large-scale nationwide study using a clinically meaningful definition of injury requiring emergency department visits or hospitalization, together with robust matching methodology and sensitivity analyses to address potential sources of bias.

In this study, we used Taiwan’s National Health Insurance Research Database (NHIRD) to investigate the association between DM and risk of clinically significant injury. We applied propensity score matching with standardized mean difference assessment to improve covariate balance and conducted multiple sensitivity analyses to evaluate robustness of findings and potential healthcare utilization bias.

## Materials and methods

### Data source

This nationwide population-based cohort study used Taiwan’s National Health Insurance Research Database (NHIRD), which contains longitudinal claims data from Taiwan’s National Health Insurance program covering more than 99% of the Taiwanese population. The NHIRD includes information on outpatient visits, emergency department visits, hospitalizations, diagnoses, procedures, and prescription medications coded according to the International Classification of Diseases, Ninth Revision, Clinical Modification (ICD-9-CM) [21, 22].

The representativeness, completeness, and validity of the NHIRD have been demonstrated in previous epidemiological studies [23]. All personal identifiers were encrypted before release to protect patient confidentiality. This study was approved by the Institutional Review Board of Tri-Service General Hospital (TSGHIRB No. E202416036), and informed consent was waived because the analysis used de-identified secondary administrative data [24].

### Study design and population

We conducted a nationwide population-based matched cohort study using Taiwan’s NHIRD. Adults aged ≥ 18 years with newly diagnosed DM between 2000 and 2013 were identified.

DM was defined using ICD-9-CM code 250, requiring either at least one inpatient diagnosis or at least two outpatient diagnoses within one year to improve diagnostic validity [25].

Individuals were excluded if they had:

1. A diagnosis of DM before cohort entry,
2. Any injury diagnosis before the index date,
3. Missing demographic information, or.
4. Age < 18 years.

For each DM patient, non-DM comparators were selected from the same database and assigned the same index date as the matched DM patient to ensure temporal comparability.

Propensity score matching was subsequently performed at a 1:3 ratio using demographic characteristics, socioeconomic variables, baseline comorbidities, healthcare utilization, and disease burden.

**Fig. 1:**
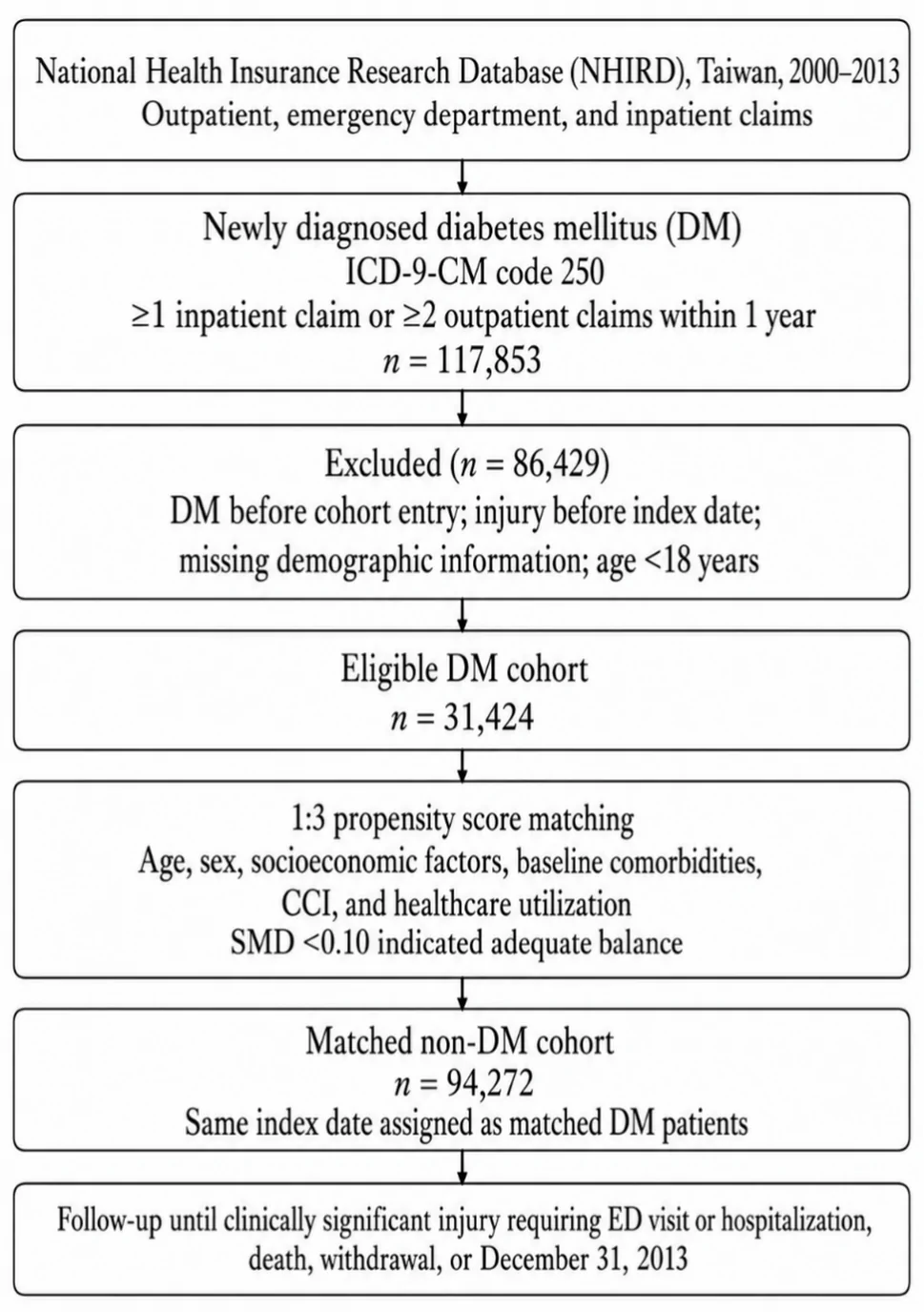
Flowchart of study cohort selection from the Longitudinal Health Insurance Database (NHIRD), Taiwan, 2000–2013

A flowchart of cohort selection is shown in Fig. 1.

Adults aged ≥ 18 years with newly diagnosed diabetes mellitus (DM) were identified from the NHIRD between 2000 and 2013. Individuals with a diagnosis of DM before cohort entry, any injury diagnosis before the index date, missing demographic information, or age < 18 years were excluded. Eligible patients with DM were matched to individuals without DM using 1:3 propensity score matching based on demographic characteristics, socioeconomic variables, baseline comorbidities, healthcare utilization, and disease burden. Non-DM comparators were assigned the same index date as the matched DM patient to ensure temporal alignment. Participants were followed from the index date until the occurrence of the primary outcome, death, withdrawal from the insurance program, or the end of follow-up on December 31, 2013.

### Exposure and index date definition

Exposure was defined as newly diagnosed diabetes mellitus (DM). DM was identified using ICD-9-CM code 250, requiring either at least one inpatient diagnosis or at least two outpatient diagnoses within one year to improve diagnostic validity [25].

The index date was defined as the first date of a qualifying DM diagnosis among exposed individuals. For each matched non-DM comparator, the same index date as the corresponding DM patient was assigned to ensure comparable follow-up initiation and temporal alignment between groups.

This design minimized immortal time bias and ensured comparable baseline risk periods between exposed and unexposed individuals [26]. All baseline covariates were assessed before the index date to preserve temporal ordering and avoid adjustment for post-exposure variables.

### Outcome definition

The primary outcome was clinically significant injury requiring emergency department (ED) visit or hospitalization. Injury events were identified using ICD-9-CM injury diagnosis codes (800–999) recorded in emergency department or inpatient claims.

Secondary outcomes included specific injury categories:

1. Traffic-related injury
2. Fall-related injury
3. Fracture
4. Burn injury
5. Poisoning
6. Intracranial or internal injury

To improve clinical specificity and reduce potential outcome misclassification, outpatient-only injury diagnoses, superficial injuries, and non-specific injury codes without emergency or inpatient healthcare utilization were excluded from the primary analysis. Broader injury definitions, including outpatient-treated injuries, were evaluated separately in sensitivity analyses [27].

The ICD-9-CM diagnosis codes and external cause codes used for the definitions of DM, comorbidities, and injury outcomes are provided in Supplementary Table S1.

### Covariates

Potential confounding variables were assessed before the index date and included demographic characteristics, socioeconomic factors, baseline comorbidities, healthcare utilization, and overall disease burden.

Demographic variables included age, sex, income level, and urbanization level.

Baseline comorbidities included hypertension, hyperlipidemia, coronary artery disease (CAD), stroke, heart failure, chronic kidney disease (CKD), chronic obstructive pulmonary disease (COPD), obesity, and obstructive sleep apnea (OSA).

Overall comorbidity burden was evaluated using the Charlson Comorbidity Index (CCI).

Healthcare utilization variables, including outpatient visits, emergency department visits, and hospitalizations before the index date, were included to reduce potential detection bias and confounding related to differences in healthcare-seeking behavior [28].

Diabetes treatment status was assessed using prescription claims for antidiabetic medications, including oral glucose-lowering agents and insulin therapy. Medication status was evaluated in secondary analyses rather than included as a matching covariate. However, information regarding medication adherence, treatment duration, dosage, and glycemic control parameters, such as glycated hemoglobin (HbA1c), was unavailable in the claims database.

### Propensity score matching and balance assessment

Propensity scores were estimated using multivariable logistic regression with DM status as the dependent variable.

Variables included in the propensity score model were selected a priori based on established epidemiological principles and previous literature regarding potential associations with both DM and injury risk. Confounders were selected according to their theoretical relationship with exposure and outcome rather than statistical significance alone [29, 30].

The propensity score model incorporated demographic characteristics (age, sex, income level, and urbanization level), baseline comorbidities (hypertension, hyperlipidemia, CAD, stroke, heart failure, CKD, COPD, obesity, and OSA), CCI, and healthcare utilization variables.

A 1:3 nearest-neighbor propensity score matching without replacement was performed using a caliper width of 0.2 standard deviations of the logit of the propensity score [29].

A 1:3 matching ratio was selected to improve statistical efficiency by incorporating additional comparable controls while maintaining adequate covariate balance. Previous methodological studies have demonstrated that increasing the number of controls can improve precision, although the incremental benefit decreases with larger matching ratios [29].

Covariate balance after matching was evaluated using standardized mean differences (SMDs). An SMD value < 0.10 was considered indicative of adequate balance between groups [30]. All measured covariates achieved satisfactory balance after matching (Table 1 and Table S1).

### Statistical analysis

Baseline characteristics were summarized using descriptive statistics. Continuous variables were presented as means with standard deviations or medians with interquartile ranges, and categorical variables were presented as numbers and percentages.

Incidence rates of clinically significant injury were calculated per 1,000 person-years. The association between DM and the risk of clinically significant injury was evaluated using Cox proportional hazards regression models.

Because individuals were selected using propensity score matching, robust sandwich variance estimators were applied to account for the matched cohort design and potential within-matched-set correlation [31]. Hazard ratios (HRs) and 95% confidence intervals (CIs) were estimated.

The proportional hazards assumption was assessed using Schoenfeld residuals.

Subgroup analyses were conducted to evaluate potential effect modification according to age, sex, socioeconomic status, and baseline comorbidities. Interaction terms between DM status and subgroup variables were tested, and p values for interaction were reported.

Among individuals with DM, exploratory analyses were conducted according to antidiabetic medication status, including oral glucose-lowering agents and insulin therapy, to evaluate whether treatment patterns modified the observed association between DM and clinically significant injury risk.

Several sensitivity analyses were performed to assess the robustness of the findings, including:

1. Exclusion of injury events occurring within 30 days after the index date.
2. Exclusion of injury events occurring within 90 days after the index date.
3. Exclusion of injury events occurring within 180 days after the index date.
4. Additional adjustment for baseline healthcare utilization variables.

All statistical tests were two-sided, and a *p*-value < 0.05 was considered statistically significant. Statistical analyses were performed using appropriate statistical software.

## Results

### Baseline characteristics and propensity score matching

After propensity score matching, 31,424 individuals with DM and 94,272 matched non-DM individuals were included in the final cohort. Baseline characteristics before and after matching are presented in Table 1 and Table S1.

After matching, all measured covariates achieved adequate balance between the DM and non-DM cohorts, with SMD below 0.10 for all variables, indicating successful reduction of baseline differences between groups.

The distributions of demographic characteristics, socioeconomic factors, baseline comorbidities, CCI, and healthcare utilization variables were comparable between the two cohorts after matching. Although age and sex were included in the propensity score model, additional covariates, including chronic diseases and healthcare utilization measures, were simultaneously incorporated to achieve overall covariate balance.

**Table 1:** Baseline characteristics before and after propensity score matching

Variable	DM (*n* = 31,424)	Non-DM (*n* = 94,272)	SMD (Before)	SMD (After)
Age (mean ± SD)	58.95 ± 13.01	58.72 ± 12.76	0.312	0.018
Male (%)	24,229 (77.10%)	24,229 (77.10%)	0.001	0.000
Hypertension (%)	16,384 (52.14%)	46,504 (49.33%)	0.398	0.056
Hyperlipidemia (%)	1,759 (5.60%)	4,460 (4.73%)	0.276	0.039
CAD (%)	5,320 (16.93%)	14,347 (15.22%)	0.301	0.047
Stroke (%)	2,779 (8.84%)	7,606 (8.07%)	0.187	0.028
CKD (%)	5,320 (16.93%)	13,870 (14.71%)	0.205	0.061
COPD (%)	3,971 (12.64%)	11,011 (11.68%)	0.132	0.029
CCI (mean ± SD)	1.85 ± 1.31	1.81 ± 1.27	0.317	0.037

Standardized mean difference (SMD) < 0.10 indicates adequate balance. After propensity score matching, all covariates were well balanced between groups

### Incidence of clinically significant injury

During follow-up, participants were observed from the index date until the occurrence of the first clinically significant injury event, death, withdrawal from the insurance program, or the end of study period. The median follow-up duration of the overall matched cohort was 1.86 years (Supplementary Table S2-1).

The mean follow-up duration was shorter in the DM cohort than in the matched non-DM cohort (2.11 ± 2.01 years vs. 3.06 ± 3.04 years, respectively). Detailed follow-up characteristics are presented in Supplementary Table S2-1.

During follow-up, clinically significant injury requiring emergency department visit or hospitalization occurred in 26,219 individuals with DM and 78,010 matched non-DM individuals. The incidence rate of clinically significant injury was higher in the DM cohort than in the non-DM cohort (123.06 vs. 112.74 per 1,000 person-years, respectively) (Table 2).

The median time from index date to the first clinically significant injury event was shorter among individuals with DM compared with matched non-DM individuals (1.41 years vs. 1.93 years, respectively) (Table S3-1 and S3-2), suggesting an earlier occurrence of medically attended injury among individuals with DM.

**Table 2:** Incidence rates of clinically significant injury requiring emergency department visit or hospitalization

Group	Individuals with injury	Person-years	Incidence rate (per 1,000 person-years)
DM	26,219	213,054.72	123.06
Non-DM	78,010	691,956.48	112.74

Clinically significant injury was defined as an injury diagnosis recorded during emergency department visits or inpatient hospitalization. Only the first qualifying injury event after the index date was considered for the primary analysis

### Association between diabetes mellitus and clinically significant injury

In the propensity score–matched cohort, individuals with DM had a higher risk of clinically significant injury requiring emergency department (ED) visit or hospitalization compared with matched individuals without DM.

Cox proportional hazards regression analysis with robust sandwich variance estimation was performed to account for the matched cohort design. After adjustment for demographic characteristics, socioeconomic factors, baseline comorbidities, CCI, and healthcare utilization variables, DM remained significantly associated with an increased risk of clinically significant injury (adjusted hazard ratio [aHR], 1.645; 95% confidence interval [CI], 1.202–2.015; *p* < 0.001).

The association remained statistically significant after accounting for potential confounding factors and differences in baseline healthcare utilization between the DM and non-DM cohorts (Table 3).

**Table 3:** Cox proportional hazards regression analysis of the association between diabetes mellitus and clinically significant injury

Model	Hazard ratio (HR)	95% CI	*p*-value
Propensity score–matched Cox model	1.645	1.202–2.015	< 0.001

The primary analysis was performed using the propensity score–matched cohort. Cox proportional hazards regression with robust sandwich variance estimators was applied to account for the matched study design. Models were adjusted for demographic characteristics, baseline comorbidities, Charlson Comorbidity Index, and healthcare utilization variables

### Subgroup analyses

Subgroup analyses were performed to evaluate whether the association between DM and clinically significant injury differed according to age, sex, and baseline comorbidities.

The association between DM and clinically significant injury remained consistent across examined subgroups, including age groups, sex categories, and hypertension status (Table 4).

No significant interactions were observed between DM status and subgroup variables (all *p* for interaction > 0.05), indicating that the association between DM and injury risk was not significantly modified by these factors.

**Table 4:** Subgroup analyses of the association between diabetes mellitus and clinically significant injury

Subgroup	Adjusted HR	95% CI	*p*-value	*p* for interaction
**Age**				
< 65 years	1.606	1.183–1.996	< 0.001	0.786
≥ 65 years	1.694	1.210–2.094	< 0.001	
**Sex**				
Male	1.722	1.340–2.188	< 0.001	0.825
Female	1.631	1.158–1.974	< 0.001	
**Hypertension**				
With hypertension	1.731	1.356–2.193	< 0.001	0.431
Without hypertension	1.622	1.146–1.970	< 0.001	

Adjusted hazard ratios were estimated using Cox proportional hazards regression models with robust sandwich variance estimators. Interaction analyses were performed to evaluate effect modification

### Sensitivity analyses

Several sensitivity analyses were conducted to assess the robustness of the association between DM and clinically significant injury (Table S4).

The association remained consistent after excluding injury events occurring shortly after cohort entry (within 30, 90, and 180 days after the index date), suggesting that the observed association was unlikely to be explained solely by early detection or reverse causation.

Additional adjustment for baseline healthcare utilization variables yielded similar risk estimates, indicating that differential healthcare-seeking behavior was unlikely to fully account for the observed association.

Furthermore, analyses excluding minor injury-related diagnostic codes and applying alternative injury definitions produced consistent results.

Overall, these sensitivity analyses supported the robustness of the association between DM and clinically significant injury requiring ED visit or hospitalization.

### Diabetes treatment status analysis

Among individuals with DM, additional analyses were conducted to evaluate whether antidiabetic medication status influenced the association between DM and clinically significant injury risk (Table S5).

Patients with DM were categorized according to prescription claims for antidiabetic medications during follow-up, including no medication use, oral glucose-lowering agents, and insulin therapy. Medication status was evaluated as an exploratory secondary analysis because treatment exposure occurred after DM diagnosis and was therefore not included as a matching covariate.

Compared with matched individuals without DM, individuals with DM remained at increased risk of clinically significant injury across all medication-status categories. The association was observed among patients without antidiabetic medication use (aHR, 1.690; 95% CI, 1.235–2.167), those receiving oral glucose-lowering agents (aHR, 1.487; 95% CI, 1.139–1.893), and those receiving insulin therapy (aHR, 1.529; 95% CI, 1.188–1.942) (Table S5).

Although the magnitude of association varied slightly according to treatment status, no medication category demonstrated complete attenuation of the increased injury risk associated with DM. These findings suggest that the observed association between DM and clinically significant injury was unlikely to be explained solely by antidiabetic medication exposure.

## Discussion

### Principal findings

In this nationwide population-based matched cohort study, individuals with DM had a significantly higher risk of subsequent clinically significant injury requiring emergency department (ED) visits or hospitalization compared with matched individuals without DM. After propensity score matching and multivariable adjustment, the association between DM and injury remained consistent, and adequate covariate balance was achieved based on SMD. These findings suggest that DM is associated with an increased risk of medically attended injury.

The revised analysis focused on clinically meaningful injury outcomes rather than broad injury definitions. Although the initial analysis using a broad ICD-9-CM injury definition demonstrated an elevated risk of injury, the high event proportion suggested that this definition may have captured minor or less clinically specific injuries. Therefore, the current findings should be interpreted as an association between DM and clinically significant injury requiring medical attention rather than evidence that DM directly causes injury.

### Comparison with previous studies and potential mechanisms

Our findings are consistent with previous epidemiological studies reporting that individuals with DM have increased risks of injury-related hospitalization, falls, fractures, and trauma-related events [32–34]. Several biological and clinical mechanisms may explain this association.

First, diabetes-related microvascular and neurological complications can impair physical function and increase injury susceptibility. Peripheral neuropathy may reduce proprioception, impair postural stability, and increase fall risk, whereas diabetic retinopathy and visual impairment may compromise environmental awareness and reaction time [35, 36]. Furthermore, hypoglycemia associated with glucose-lowering medications, particularly insulin and insulin secretagogues, may impair attention, cognitive performance, and psychomotor function, thereby increasing the risk of falls and traffic-related injuries [37].

Second, DM is frequently accompanied by multiple chronic conditions, including cardiovascular disease, chronic kidney disease, and cerebrovascular disease. These comorbidities may contribute to frailty, reduced physiological reserve, impaired mobility, and increased vulnerability to injury, particularly among older adults [38, 39]. In addition, diabetes-associated muscle dysfunction and accelerated loss of skeletal muscle strength may further increase the risk of falls and fractures [40].

### Injury patterns and clinical relevance

Falls and traffic-related injuries represent important causes of preventable morbidity among individuals with chronic diseases. Previous studies have demonstrated that DM is associated with increased risks of falls, fractures, and motor vehicle collisions through mechanisms involving impaired vision, peripheral neuropathy, hypoglycemia, cognitive dysfunction, and reduced physical function [41–43].

The present findings emphasize the importance of integrating injury prevention into routine diabetes care. Beyond glycemic control, clinicians should consider injury-related risk assessment as part of comprehensive chronic disease management, particularly among patients with advanced age, diabetes complications, or multiple comorbidities.

Preventive strategies may include assessment of fall risk, evaluation of vision and peripheral neuropathy, medication review to minimize hypoglycemia risk, and interventions targeting physical function and mobility.

### Sex-specific association between diabetes mellitus and injury risk

In this study, the association between DM and clinically significant injury was observed in both males and females, although the magnitude of association differed slightly between sexes [44–48]. Although DM was associated with increased injury risk in both males and females, the magnitude of association may vary by sex. Previous injury epidemiology studies have generally reported higher injury rates among males, particularly for trauma-related events, occupational injuries, and traffic-related injuries [44, 45]. Therefore, the observed sex-related pattern in our study warrants further consideration.

Several potential mechanisms may explain these findings. First, sex differences in diabetes-related complications and aging-related vulnerability may influence injury susceptibility. Females with DM, particularly older women, may have increased vulnerability to falls and fractures because of reduced muscle strength, impaired balance, osteoporosis, and frailty. Diabetes-related peripheral neuropathy and visual impairment may further increase functional limitations and susceptibility to injury [46, 47].

Second, differences in injury patterns may contribute to sex-specific risk profiles. Although males generally have greater exposure to occupational hazards, hazardous activities, and high-risk trauma mechanisms, females, particularly older adults with chronic diseases, may experience a greater burden of fall-related and fracture-related injuries due to reduced physical reserve and skeletal fragility [46, 47].

Third, differences in healthcare-seeking behavior may influence injury ascertainment in claims-based studies. Females are generally more likely to seek medical attention after health-related events, which may increase the likelihood of injury diagnosis and documentation within administrative healthcare databases [48]. Therefore, observed sex-related differences should be interpreted cautiously, as they may reflect a combination of biological susceptibility, injury patterns, and healthcare utilization behaviors rather than differential biological effects alone.

Further studies incorporating detailed information on physical activity, frailty status, bone health, environmental exposure, injury mechanisms, and injury severity are needed to clarify sex-specific injury risk among individuals with DM.

### Healthcare utilization bias and interpretation of comorbidity findings

Healthcare utilization bias represents an important consideration in claims-based observational studies. Patients with DM generally have more frequent healthcare encounters, including outpatient visits and chronic disease monitoring, which may increase the probability that injuries are detected and recorded.

Although the revised analysis adjusted for baseline healthcare utilization and included sensitivity analyses restricted to clinically significant injuries requiring ED visits or hospitalization, residual surveillance bias cannot be completely excluded.

The observed lower injury risk among patients with certain comorbidities should not be interpreted as a biological protective effect. Instead, this finding may reflect differences in healthcare engagement, preventive care, disease monitoring, activity patterns, or competing risks. Individuals with multiple chronic diseases often have more frequent healthcare contact, which may facilitate earlier identification and management of risk factors but may also introduce differential detection patterns in claims-based studies.

Therefore, these findings should be interpreted as potential indicators of healthcare utilization patterns rather than direct protective effects of comorbid diseases [49].

### Clinical implications

The findings of this study have important implications for clinical practice. Injury prevention should be incorporated into comprehensive diabetes management, particularly among older adults and patients with diabetes-related complications.

Current diabetes care recommendations emphasize individualized treatment strategies, prevention of hypoglycemia, and systematic assessment of diabetes complications that may impair physical function [50].

Potential preventive strategies include medication optimization, evaluation of hypoglycemia risk, assessment of vision impairment and peripheral neuropathy, routine fall-risk screening, and interventions aimed at maintaining mobility and functional independence. Diabetes is associated with increased disability and functional decline among older adults, highlighting the importance of preventing injury-related complications [51, 52].

Older adults with DM are particularly vulnerable because aging-related frailty, reduced muscle strength, impaired balance, and multimorbidity may further increase injury risk [53, 54]. Therefore, comprehensive geriatric assessment and individualized rehabilitation strategies may be beneficial.

Fall prevention interventions, including exercise training, environmental modification, balance improvement, and medication review, have been recommended to reduce fall-related morbidity among older adults [55–58]. Incorporating these approaches into diabetes management may reduce preventable injury events.

### Strengths and limitations

This study has several strengths. First, the use of a nationwide population-based database with extensive healthcare coverage provided a large sample size and enhanced generalizability within Taiwan. The National Health Insurance Research Database (NHIRD) has been widely validated and applied in epidemiological research, supporting the reliability of claims-based studies [59, 60].

Second, the matched cohort design established temporal sequencing between DM diagnosis and subsequent injury occurrence. Third, the revised analytical framework incorporated propensity score matching, standardized mean difference assessment, and sensitivity analyses to improve methodological rigor. Propensity score methods are widely recommended for reducing confounding in observational studies by improving comparability between exposed and unexposed groups [57]. Furthermore, SMD is considered an appropriate method for evaluating covariate balance after matching [58].

Several limitations should also be acknowledged. First, because this study relied on administrative claims data, misclassification of DM, comorbidities, and injury outcomes is possible. However, the use of validated diagnostic coding algorithms may reduce this concern.

Second, several clinically important variables were unavailable, including HbA1c levels, diabetes duration, body mass index, physical activity, frailty status, alcohol consumption, occupational exposure, environmental factors, and detailed injury severity.

Although prescription claims allowed identification of antidiabetic medication use, including oral glucose-lowering agents and insulin therapy, information regarding medication adherence, treatment duration, glycemic control, and diabetes severity could not be fully assessed. Therefore, residual confounding related to diabetes severity may persist despite propensity score matching and adjustment.

Third, healthcare utilization differences between individuals with and without DM may contribute to detection bias. Although baseline healthcare utilization variables were incorporated and sensitivity analyses were performed, residual surveillance bias cannot be completely excluded.

Finally, because this was an observational study based on secondary administrative data, causal inference cannot be established. The findings should therefore be interpreted as an association between DM and clinically significant injury risk rather than evidence of direct causation.

## Conclusion

In this nationwide population-based matched cohort study, diabetes mellitus was associated with an increased risk of clinically significant injury requiring emergency department visits or hospitalization. After propensity score matching and adjustment for baseline characteristics, the association remained consistent, suggesting that individuals with DM represent a population at increased risk of medically attended injury.

The findings highlight the importance of incorporating injury risk assessment into comprehensive diabetes management, particularly among older adults and individuals with diabetes-related complications, frailty, or functional impairment. Preventive strategies, including hypoglycemia risk assessment, medication optimization, fall-risk evaluation, and management of diabetes complications, may help reduce preventable injury-related morbidity.

Because this study was based on observational administrative data, causal relationships cannot be established. Further studies incorporating detailed clinical information, including glycemic control, diabetes severity, medication adherence, and functional status, are needed to clarify the mechanisms underlying the association between DM and injury risk.

## Supplementary Information

Supplementary Material 1.

## Data Availability

The data that support the findings of this study are available from NHIRD, but restrictions apply to the availability of these data, which were used under license for the current study, and so are not publicly available. Data are however available from the corresponding author upon reasonable request and with permission of NHIRD.
