# Supplementary material for: Diabetes mellitus and risk of injury requiring medical care: a nationwide population-based matched cohort study in Taiwan
Source: Inj Epidemiol. 2026 Aug 5;13:70. doi: 10.1186/s40621-026-00703-3 (PMC13726205; doi:10.1186/s40621-026-00703-3)
Supplement: Supplementary file 1 [file 40621_2026_703_MOESM1_ESM.docx]

**Table S1. Abbreviation, ICD-9-CM, ATC-Code, and definition**

| **Variables** | **Abbreviation** | **ICD-9-CM / ATC-Code / Definition** |
| --- | --- | --- |
| **Study population:** | | |
| **Diabetes mellitus** | DM | 250; ≧3 outpatient visits or inpatient in 1 year |
| Medication |  | A10, used ≧ 90 days |
| Oral glucose-lowering agents |  | A10B, used ≧ 90 days |
| Insulin therapy |  | A10A, used ≧ 90 days |
| **Events:** Injury |  | 800-999 |
| **Injury diagnosis** | | |
| Head injury |  | 800–804, 850–859, 873, 910, 920, 951 |
| Fracture |  | 800–829 |
| Dislocation |  | 830–839 |
| Sprains and strains |  | 840–849 |
| Intracranial / internal injury |  | 850–869 |
| Open wound |  | 870–899 |
| Injury to blood vessels |  | 900–904 |
| Superficial injury / contusion |  | 910–924 |
| Crushing |  | 925–929 |
| Foreign body entering through orifice |  | 930–939 |
| Burn |  | 940–949 |
| Injury to nerves and spinal cord |  | 950–957 |
| Poisoning |  | 960–989 |
| **Cause of injury** |  |  |
| Unintentional injury |  | E800-E949 |
| Traffic injuries |  | E800-E849 |
| Poisoning |  | E850-E869 |
| Falls |  | E880-E888 |
| Burns and fires |  | E890-E899 |
| Drowning |  | E910 |
| Suffocation |  | E911-E915 |
| Crushing / cutting / piercing |  | E916-E920 |
| Excessive heat |  | E900 |
| Injury caused by animal |  | E906 |
| Electric current injury |  | E925 |
| Other unintentional injuries |  | E870-E879, E901-E905, E907-E909, E921-E924, E926-E949 |
| Intentional injury |  | E950-E979, E990-E999 |
| Suicide |  | E950-E959 |
| Homicide / abuse |  | E960-E969 |
| Intention unknown |  | E980-E989 |
| **Comorbidities** | | |
| Hypertension | HTN | 401.1, 401.9, 402.10, 402.90, 404.10, 404.90, 405.1, 405.9 |
| Hyperlipidemia |  | 272 |
| Obesity |  | 278 |
| Macrovascular complications of DM |  |  |
| Stroke |  | 430-438 |
| Peripheral arterial disease | PAD | 440.2-440.3, 440.8-440.9, 443, 444.22, 444.8, 447.8- 447.9 |
| Coronary artery disease | CAD | 410-414 |
| Chronic obstructive pulmonary disease | COPD | 490-496 |
| Heart failure | HF | 428 |
| Chronic kidney disease | CKD | 250.4, 274.1, 403-404, 440.1, 442.1, 447.3, 572.4, 580-589, 642.1, 646.2, 753 |
| Obstructive sleep apnea | OSAs | 780.51, 780.53, 780.57 |

**Table S2. Propensity score model and covariate balance assessment**

**Propensity score model (logistic regression)**

Outcome variable:

- Diabetes mellitus (DM: yes vs no)

**Covariates included in propensity score model**

| **Category** | **Covariate** |
| --- | --- |
| Demographics | Age |
| Demographics | Sex |
| Socioeconomic | Income level |
| Socioeconomic | Urbanization level |
| Comorbidities | Hypertension |
| Comorbidities | Hyperlipidemia |
| Comorbidities | Coronary artery disease |
| Comorbidities | Stroke |
| Comorbidities | Heart failure |
| Comorbidities | Chronic kidney disease |
| Comorbidities | COPD |
| Comorbidities | Obesity |
| Comorbidities | Obstructive sleep apnea |
| Health service use | Outpatient visits (baseline) |
| Health service use | Emergency visits (baseline) |
| Health service use | Hospitalizations (baseline) |
| Comorbidity burden | Charlson Comorbidity Index (CCI) |

**Covariate balance assessment (Standardized Mean Differences)**

| **Variable** | **SMD Before Matching** | **SMD After Matching** |
| --- | --- | --- |
| Age | 0.312 | 0.018 |
| Sex | 0.001 | 0.000 |
| Income level | 0.145 | 0.032 |
| Urbanization | 0.221 | 0.041 |
| Hypertension | 0.398 | 0.056 |
| Hyperlipidemia | 0.276 | 0.039 |
| Coronary artery disease | 0.301 | 0.047 |
| Stroke | 0.187 | 0.028 |
| Heart failure | 0.164 | 0.033 |
| CKD | 0.205 | 0.061 |
| COPD | 0.132 | 0.029 |
| Obesity | 0.118 | 0.022 |
| OSA | 0.101 | 0.019 |
| Outpatient visits | 0.288 | 0.044 |
| ER visits | 0.244 | 0.038 |
| Hospitalizations | 0.261 | 0.051 |
| CCI | 0.317 | 0.037 |

**Note:**

Standardized mean differences (SMDs) were used to assess covariate balance between groups, with values <0.10 indicating adequate balance after propensity score matching. After matching, all covariates demonstrated satisfactory balance.

Propensity score matching was performed using a 1:3 nearest-neighbor algorithm without replacement and a caliper width of 0.2 standard deviations of the logit of the propensity score.

**Table S3-1. Years of follow-up**

| **DM** | **Min** | **Median** | **Max** | **Mean ± SD** |
| --- | --- | --- | --- | --- |
| With | 0.01 | 6.32 | 13.99 | 6.78 ± 4.03 |
| Without | 0.01 | 6.87 | 13.99 | 7.34 ± 4.11 |
| Total | 0.01 | 6.68 | 13.99 | 7.20 ± 4.16 |

**Table S3-2. Years to external causes of injury and poisoning**

| **DM** | **Min** | **Median** | **Max** | **Mean ± SD** |
| --- | --- | --- | --- | --- |
| With | 0.01 | 2.54 | 13.12 | 3.14 ± 2.89 |
| Without | 0.01 | 3.01 | 13.81 | 3.99 ± 3.07 |
| Total | 0.01 | 2.88 | 13.81 | 3.78 ± 3.05 |

**Table S4. Sensitivity analyses for the association between diabetes mellitus and clinically significant injury**

| **Analysis** | **Adjusted HR** | **95% CI** | ***p*-value** |
| --- | --- | --- | --- |
| Primary analysis | 1.645 | 1.202–2.015 | <0.001 |
| Excluding events within 30 days after index date | 1.622 | 1.184–1.983 | <0.001 |
| Excluding events within 90 days after index date | 1.605 | 1.167–1.925 | <0.001 |
| Excluding events within 180 days after index date | 1.583 | 1.103–1.876 | <0.001 |
| Additional adjustment for baseline healthcare utilization | 1.638 | 1.197–2.003 | <0.001 |

**Note:**

Sensitivity analyses were performed to evaluate the robustness of the association between diabetes mellitus and clinically significant injury. The association remained consistent after excluding early injury events after cohort entry, adjusting for baseline healthcare utilization, and excluding minor injury-related diagnoses

**Table S5. Association between diabetes mellitus and clinically significant injury according to antidiabetic medication status**

| **Diabetes treatment status** | **Events** | **Adjusted HR** | **95% CI** | ***p*-value** |
| --- | --- | --- | --- | --- |
| DM without medication | 10,214 | 1.690 | 1.235–2.167 | < 0.001 |
| DM with oral glucose-lowering agents | 7,972 | 1.487 | 1.139–1.893 | < 0.001 |
| DM with insulin therapy | 8,033 | 1.529 | 1.188–1.942 | < 0.001 |

**Note:**

Antidiabetic medication status was identified based on prescription claims for oral glucose-lowering agents and insulin therapy in the National Health Insurance Research Database. Medication status was evaluated as an exploratory secondary analysis among individuals with diabetes mellitus and was not included as a matching covariate because treatment exposure occurred after diabetes diagnosis. Hazard ratios (HRs) were estimated using Cox proportional hazards regression models with robust sandwich variance estimators to account for the matched cohort design. Models were adjusted for demographic characteristics, socioeconomic factors, baseline comorbidities, Charlson Comorbidity Index, and baseline healthcare utilization. Because medication adherence, treatment duration, dosage, glycemic control (e.g., glycated hemoglobin levels), and diabetes severity were unavailable in the claims database, residual confounding related to treatment intensity and disease severity may remain.

Because medication initiation may reflect underlying diabetes severity, these findings should be interpreted as exploratory associations rather than causal effects of antidiabetic treatment.
